# In the Patented Bag: Peanuts, Packaging, and Intellectual Property in the United States, 1906-1932

**DOI:** 10.1017/eso.2023.21

**Published:** 2023-09-11

**Authors:** Tad Brown

**Affiliations:** Department of History and Philosophy of Science, University of Cambridge, United Kingdom

## Abstract

This article explores the early history of two American peanut companies: Planters and Tom’s. Both food manufacturers developed major commercial brands through the ownership of intellectual property. In this case, the sourcing of different peanut types figured into the marketing of salted peanuts. Through a legal dispute involving Tom’s patented retail bag, I examine how food packaging changed how peanuts were advertised, distributed, and consumed. The argument is made for an historical analysis of food brands that considers how intellectual property domains interacted with each other and with the material properties of the food itself.

## In the Beginning

Entering the twentieth century, transportation in the United States had redefined the distance of food sources. Meats and fruits arrived by steamship or rail from far-off places. Also, industrial processing and refrigeration added shelf-life to perishable items, an annihilation of time to accompany that of space.^1^ Scholars have noted how the alienation of industrial society privileged the trust and persuasion afforded by food packaging.^2^ The era of suited salesmen yielded to promotional print as corporations found new ways to appease public worries about the convenience of processed foods.^3^ Packaging and advertising were joint features of modern food marketing.

Manufacturers selling similar foods at nearly-identical prices sought to differentiate their mass-produced items with brand labels. The legal protection afforded to trademark—a type of intellectual property that identifies a commercial source through an exclusive name or image—played a prominent role in the development of brands. Irreducible to insignia, a brand can be understood as a “product narrative”, a social phenomenon of identifying goods with symbolic meaning and desires.^4^ Scholars interested in branding have given disproportionate attention to firm-specific studies from the food and beverage industry, which David Higgins attributes to “the transformation of their famous trademarks into valuable brands.”^5^ This article adds to that body of work by examining two American peanut firms, Planters and Tom’s. I argue that trademark was important to peanut branding for both companies, but only in conjunction with other domains of intellectual property.

Planters and Tom’s used various types of intellectual property to establish their businesses in the early twentieth century. The US Patent & Trademark Office (USPTO) oversaw the application and registration of patents and trademarks. While trademark protection endures with commercial use, patent rights (at the time) expired after seventeen years from date of grant. Trade secrets were unique insofar as protection was achieved through a lack of disclosure; exclusivity for trade secrets was not issued by the USPTO, but rather held in confidence by its possessors. The “product narrative” of salted peanuts sold by Planters and Tom’s involved some combination of these different types of intellectual property protections.^6^

Business historians have thoroughly examined the role of branding in the shift to modern consumerism.^7^ Paul Duguid argued that, contrary to the typical interpretation by historians, who situate the rise of modern branding among vertically-integrated twentieth-century corporations seeking to distinguish their goods from commercial rivals, earlier examples of branding involve legal disputes between various actors in the *same* supply chain.^8^ With evidence from a study of alcohol in the nineteenth century, Duguid challenged the consumer-facing orthodoxy of brands. His proposal that branding exercises control over supply relations may have particular significance for the industrial food system. Early agribusiness was marked by a lack of total vertical integration, resulting in a context where power concentrated at specific points along the supply chain.^9^ In particular, the industrial agricultural system afforded commercial food processors substantial leverage in mediating the delivery of packaged foods. Many of these same processors became household names, including Planters and Tom’s.

By the 1910s, advertising agencies in the United States began to actively position trademarks within strategic campaigns to construct brand images.^10^ Art historian Carma Gorman has questioned the tendency of scholars to reference avant-garde advertising firms as an explanation for the development of corporate brands. For Gorman, the company-biographical mode draws a questionable connection between personal narratives and later developments in the field of advertising.^11^ Gorman, instead, posited that legal changes in the United States persuaded businesses to invest in visual identity branding. Given this reasoning, the structure of trademark law, not the ingenuity of individuals, is made responsible for changes in advertising.

There is a need for studies that explore the relationship between intellectual property and food history, especially with a focus on brand advertising, product materiality, and retail delivery. To the extent that intellectual property has featured in the historical analysis of food and agriculture, the emphasis has overwhelmingly been on seeds.^12^ The literature has focused on the ownership of crop varieties, whereas prior developments across the food supply chain are equally important for historicizing the seed industry and intellectual property in plants. This article presents evidence that, at least with peanuts, food manufacturing companies in the early twentieth-century leveraged various domains of intellectual property protection to conform crop varieties—through the selection and distribution of seedstocks—to their own brand standards, prior to such crop varieties becoming eligible for intellectual property ownership.^13^ This marketing strategy appeared in mass advertising and product labelling.

Various scholars have shown how adding labels to foodstuff facilitated a redefinition of food via visual cues, regulatory standards, and social stigmas.^14^ Xaq Frohlich stated, for example, that nutrition labels served as part of the “information infrastructure” that organized how food was produced, distributed, and consumed.^15^ By positioning packaging within this information infrastructure—as the square-inches on which product labels circulate—I consider how intellectual property became indispensable to peanut sales in the United States. What had been the buying and selling of an agricultural commodity by jobbers, who delivered generic produce for the wholesale trade, became a capital investment into consumer brands.

Both Planters and Tom’s owned the capital infrastructure to clean, sort, grade, and shell peanuts (*Arachis hypogaea*) from nearby crop fields. Each company also manufactured a line of labelled food products. The geography of peanut agriculture in the United States became embedded in these respective retail brands. Planters wed its brand identity to Virginia-type peanuts, a commercial class of large peanuts grown almost exclusively in the tidewater counties of the Virginia-Carolina region.^16^ Similarly, Tom’s built its commercial empire on the Small White Spanish peanut, the standard variety grown in the Deep South peanut-belt, located at the confluence of Georgia, Florida, and Alabama. Planters and Tom’s used the ownership of intellectual property to differentiate their packaged products in relation to these specific peanuts.

In what follows, I historicize developments in the U.S. peanut industry during the early twentieth century. The Planters Nut & Chocolate Company helped establish a nationwide market for salted peanuts. Few manufacturers had the purchasing power or owned the intellectual property to imitate Planters’ commercial success, though some tried. With the Tom Huston Peanut Company, the flattery was returned in kind. A patented retail bag brought Tom’s nationwide sales in the mid-1920s, and led to numerous legal disputes, including a case involving Planters. I review the patent litigation to show how trademark relied on other domains of intellectual property for brand development. The conclusion draws together the establishment of commercial brands with changing supply relations in the industrial food system.

## Planters Peanuts’ Past

Virginia was the birthplace of the American peanut industry. As a “meeting-point of five railroads,” the five-story tall factories in Suffolk, and nearby Norfolk, combined to clean over half of the country’s crop in the early 1900s.^17^ Trade activity in Suffolk effectively established the price received by peanut growers in the rest of the country. The city was commonly referred to as the “World’s Greatest Peanut Center.”^18^ Commercial trends in the United States’ peanut trade originated from shelling companies in Suffolk, with Planters becoming the most famous. The company moved to Suffolk eventually, but it started elsewhere.

Planters was founded in Wilkes-Barre, Pennsylvania by two Italian immigrants, Amedeo Obici (1877-1947) and Mario Peruzzi (1875-1955). Obici had sailed for America from his hometown on the outskirts of Venice and started peddling peanuts from a fruit cart in Wilkes-Barre at age twelve. He quickly found a following selling salted peanuts and dubbed himself “The Specialist,” a name he had trademarked and emblazoned in gold-letters on his horse-drawn wagon.^19^ Not only was Obici credited as the first peanut peddler to sport a whistle, signalling to passers-by within earshot, he put one of six letters inside each manila bag of peanuts, gifting a complimentary watch to anyone who spelled the magic word “AOBICI.”^20^ Salesmanship would be a mainstay of Planters’ success.

Similar to other peanut sellers in the region, Obici “confined himself chiefly to the unshelled variety.”^21^ Selling whole roasted jumbo Virginia peanuts in the shell was in vogue. Obici would soon deviate from this trend to “shell, salt, and dress up the peanut,” separating his product from other generic offers.^22^ As the story goes, one fateful day Obici met his would-be partner Peruzzi in a grocery store where the latter worked. With limited capital, the two immigrants founded Planters Peanuts in 1906. They incorporated two years later as the Planters Nut & Chocolate Company, with Obici as President and Peruzzi the Secretary-Treasurer.^23^

The immigrant merchants struggled to turn a profit at first. There was an obvious reason for the sustained deficit. The owners had decided to source, shell, and sell the Extra-Large Virginia peanuts, instead of “the small round red-skinned Spanish nuts that dominated the market” for shelled peanuts (without shell).^24^ Abandoning the cheaper standard came with economic consequences. Planters compensated for the higher price-point of the Virginia-type peanut by bagging their product in smaller packages, compared to the quantity of Spanish kernels received for the same price by other vendors. The change in packaging had an incidental upside.

Deterioration of product quality was a problem with many bulk edibles, and none more so than peanuts. The paper bags employed in the industry could not keep a hermetic seal, so regardless of portion size, salted peanuts attracted moisture from the atmosphere, hastening the rate at which the snack went stale. Even worse, peanut oils oxidized with exposure to air and imparted a rancid flavour. Obici wondered what could be done to prevent the unrecoverable losses to spoilage. Packaging design offered one solution. In 1908, the USPTO granted Obici a patent for “a double bag for the vending of salted peanuts.”^25^ The invention consisted of an inner package made of moisture-proof material which increased product shelf-life. The extent of its manufacture would later become a point of legal dispute. Nonetheless, Planters’ willingness to experiment with retail packaging testifies to the early potential of intellectual property to remake the peanut trade.

In 1913, Obici and Peruzzi decided to relocate operations to Suffolk, Virginia.^26^ The owners purchased a peanut cleaning facility and, in time, expanded their commercial properties in Suffolk to include cold storage, warehouse space, and printing and packaging facilities.^27^ The move by Planters reduced the costs involved in sourcing Virginia peanuts. Peruzzi would later state: “Because of the high price of the best grade of peanuts we were forced to go down into the Virginia fields and raise our own crops. We were told by the people in Suffolk that we had more money than brains … that we did not know anything about peanuts.”^28^ To what extent Planters actually produced peanuts is unknown, but the facilities in Suffolk allowed the manufacturer to buy direct from nearby producers “without the expense of long hauls of the unfinished product.”^29^

This approach at vertical integration had precedents in the food industry. For instance, the Heinz Company of Pittsburgh, Pennsylvania adopted a similar strategy in the late nineteenth century. Heinz manufactured its own supply of vinegar for pickling and acquired thousands of acres of farmland to supervise crop production.^30^ Likewise, the Hershey Chocolate Company purchased a sugar plantation in Cuba.^31^ Control over ingredients brought assurance during a time of heightened distrust about food purity.^32^ In this way, the relocation to Suffolk facilitated Planters’ access to and oversight of Extra-Large Virginia peanuts.

Close proximity to peanut fields was an element of Planters’ success, an advantage shared by other companies in the area. What Obici and Peruzzi did that was unique was recondition public opinion through a relentless commitment to advertisement.^33^ The company popularized the slogan “The Nickel Lunch” to help categorize peanuts as a substantial food, and not merely a confectionary item. Most peanut jobbers at the time displayed salted peanuts in large glass jars with an accompanying supply of manila bags.^34^ Planters did too. But around 1910, Planters adopted glassine paper bags, which, as a semi-transparent precursor to cellophane, enabled the company to package sealed envelopes of salted nuts for retail. The company introduced vacuum-sealed jars made of blue tin in the 1920s.^35^ War rationing eventually forced Planters to substitute metal with glass, but its line of “Cocktail Peanuts” persisted as a staple brand of the snackfood industry.

Regardless of the choice of packing material, evidence of Planters’ intellectual property appeared on each individual item. When *Modern Packaging* published an article on Planters, the magazine identified “one of the most important single factors in the development of the Planters Company, aside from packaging, was the creation of Mr. Peanut.”^36^ In 1917, Planters introduced its logo Mr. Peanut. Obici paid five-dollars to a local Italian-American schoolboy who submitted the winning entry to a sponsored contest. The president promptly hired a professional artist to elaborate on the original sketches.^37^ The anthropomorphized peanut with a monocle, silk top hat, English cane, gloves and pied spats became a distinct industry icon. By 1921, Mr Peanut was “known everywhere in the broad land of America.”^38^ Planters paid for advertisements in the pages of *Saturday Evening Post, Good Housekeeping*, and other leading periodicals.^39^ The company began to turn a steady profit two decades after its founding.

To believe historical boosters from Virginia, few people had recognized the possibilities of peanuts prior to Planters.^40^ Peanuts were long perceived as “slave food” in the United States.^41^ Though domesticated in South America, the crop was introduced from West Africa aboard slave ships, and the agrarian geography of peanut production confined the crop to slave-holding colonies throughout the 18^th^ century.^42^ Black people grew peanuts in small food plots, with a few households in Virginia including the plant in their gardens “almost as curiosities.”^43^ Only after Union soldiers gained familiarity with peanuts during the Civil War did the crop enter into interstate commerce. Circusgoers and pedestrians throughout the country took to buying peanuts, being viewed as something “a few farmers planted and … a few lowly men and churlish lads cracked and crunched.”^44^ This was the status of *Arachis* at the turn of the century, a racialized food of lower-class repute.

Sensing opportunity, food manufacturers ventured to capitalize on the changing demographic of the peanut-eating market. Of all, Planters displayed a special knack for remaking the snack’s image and furnishing the convenience food with a new narrative. *The Suffolk News-Herald* offered this hyperbolic praise: “In Planters we have another illustration of the power of an idea, which, when guided by a master hand, develops into tangible existence to gladden him in whose mind it was born and those whose lives it touches.”^45^ Selling salted peanuts by their intangibles was a way to disassociate the crop from its historical roots. Yet even this marketing approach depended on the physical properties of the peanuts themselves.

Central to Planters’ brand and its trademark logo was the allure imparted by a trade secret. At his very first public appearance, Mr. Peanut boasted about a “secret, private process” for producing a whole blanched salted peanut.^46^ Obici had developed a method for keeping the peanut intact during the removal of peanuts skins, known as blanching. Exact details of the process were never disclosed, only its vague origins: “We discovered the process for keeping the peanut whole while preparing salted peanuts.”^47^ Other manufacturers also sold nickel bags of salted peanuts, but unlike Planters, their peanuts came halved. The trade secret gave Planters a product with a unique appearance. The “scarcity of splits” became “one of the marks of distinction” for Planters’ brand.^48^ Products sold with the image of Mr. Peanut exhibited a tangible quality that was absent in other offerings of Extra-Large Virginia peanuts.

Planters advertised this visual difference as a way to make social distinctions. Throughout the first year of the Mr. Peanut marketing campaign, the logo appeared in print media to promote the brand. The public was repeatedly reminded that Planters peanuts were the pick of the crop, “prepared by our private process.”^49^ One newspaper advertisement showcased Mr. Peanut at a “nice dinner party” offering a platter of peanuts to three uppity guests. The copywriting read: “They’re not like the kind of salted peanuts you’ve been used to. No, sir. They’re *whole*, and big, and appetizing *looking*.”^50^ Planters branding strategy, reassuring white society of its general respectability, was commonplace in the first two decades of the twentieth century.^51^ The object was to gentrify peanut consumption. The visual appeal of Planters’ peanuts allowed the company to sell their product as comparable to almonds.

**Figure 1:**
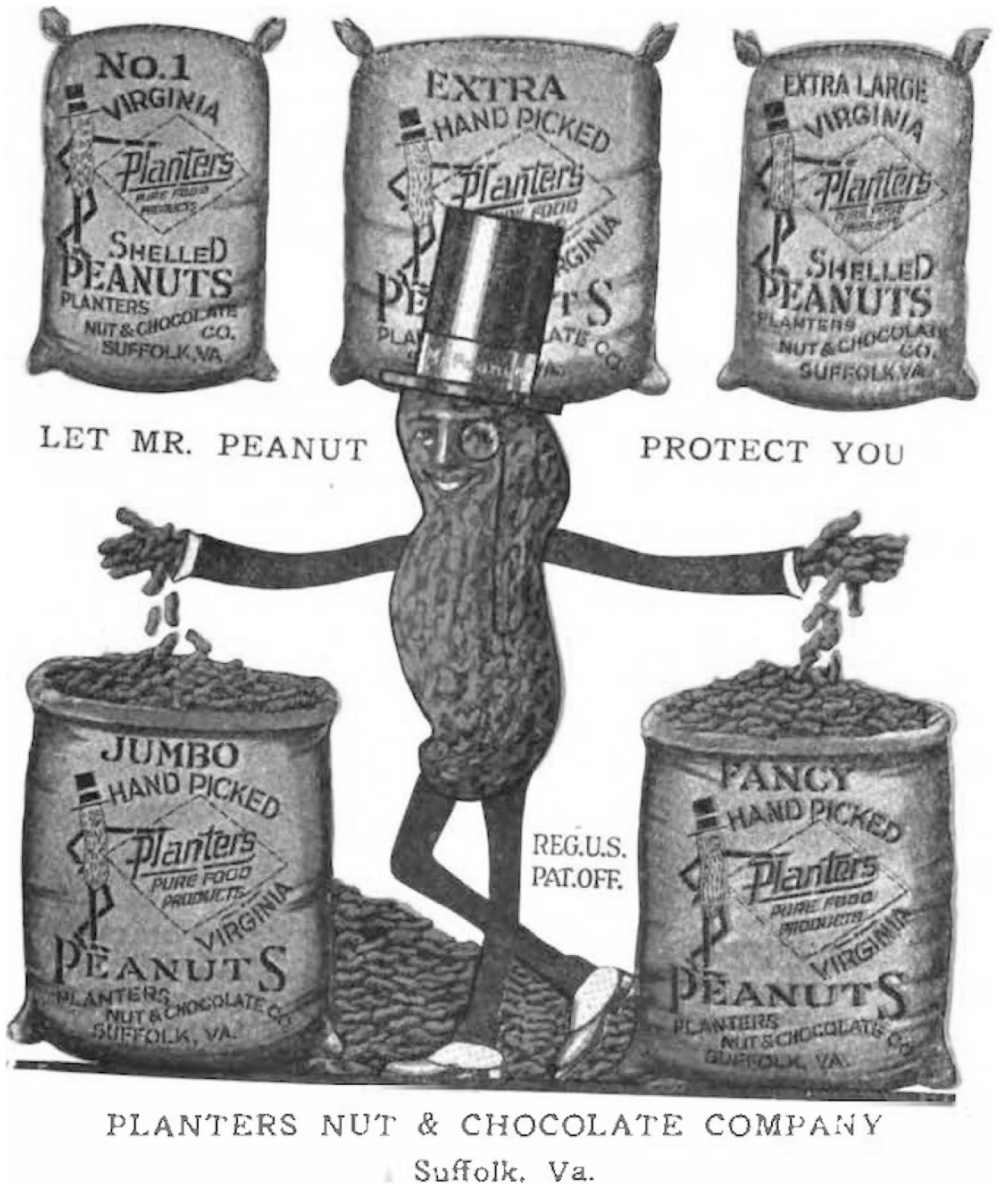
Mr Peanut on the cover of an industry periodical, advertising Planters as a regional peanut buyer. Source: *The Peanut Promoter* 7, no. 3 (1922)

Obici and Peruzzi registered their trademark in 1918, which granted the logo nationwide protection.^52^ The aristocratic figure appeared on every retail package that Planters sold. Mr. Peanut’s image also donned the burlap sacks of peanut seed bought by the company. [See Fig 1] That Planters’ trademark accompanied the movement of peanuts from harvest to retail supports Duguid’s contention that branding influenced vendor relations within the same supply chain. Interestingly, Duguid takes issue with business histories that attribute the rise of modern branding to packaging because of the technological determinism in that retelling. What Planters’ history suggests is that packaging might not matter as much as the intellectual property it conveyed. Plus, by marketing its brand in reference to the visual attributes of the Virginia-type peanut, the company generated demand for agricultural produce from the greater Suffolk region.^53^ Planters supported local growers with allegiance to the peanut type, a point of pride emphasized in company advertisements.

## Call Him Tom

The peanut industry in the Deep South experienced several developments as a result of World War I, including a number of shelling plants. Among those plants constructed after the war was that of the Tom Huston Peanut Company in Columbus, Georgia. Unlike Planters, Tom’s built an empire on the Small White Spanish peanut, “a crop to which the farmers of that section turned with hope after the ravages of the boll weevil had made the raising of cotton so unprofitable.”^54^ Small White Spanish peanuts were the standard commercial type sold to oil mills as a substitute for cottonseed, yet Tom had other ideas for the oleaginous nut.

John Thomas “Tom” Huston (1889-1972) came of age on a peanut farm in the sand hills of east Texas. Shelling peanuts by hand wore his fingers raw and inspired Huston’s first invention.^55^ In July 1915, he filed a patent application for a hand-crank peanut shelling machine. By adding a movable concave grate, the sheller could thresh with “minimum breakage.”^56^ (Allegedly, he found inspiration for the design from a similar machine for shelling corn.) Huston soon moved to Columbus, Georgia and signed an agreement with the Columbus Iron Works to manufacture his shellers. An advertisement from 1916 claimed, “The Tom Huston Peanut Sheller is the only successful machine on the market.”^57^ Sales were encouraging.^58^ Machines by the Tom Huston Manufacturing Company soon operated in shelling plants across the southern United States.^59^

Processors in the peanut industry held regular dialogue with farmers, and their shared complaints led to new inventions. In April 1922, the USPTO granted Tom Huston a patent on a peanut digger with “a peculiar type of blade.”^60^ The scope of the patent was not limited to the V-shaped blade, but its design specifically addressed problems with digging the taproot of Spanish peanuts. Huston was granted this patent while still manufacturing peanut shellers, yet his active interest in how peanuts were grown carried over into his career as a food processor. The restless inventor found that selling quality machines resulted in few repeat customers. So, Huston decided to sell his interest in the manufacturing company and relocate to a two-room wooden shack in Columbus to start afresh. He began packing salted peanuts, in 1925, with a trademark upside-down red triangle.^61^

The nickel bag of peanuts was nothing new as Planters’ preceding history attests. Tom’s Toasted Peanuts made its mark, however, by possessing a special flavor, one attributed to the Small White Spanish peanut and a special method of manufacture. According to an observer, “The Huston plant has made salted peanuts taste like they have never tasted before; by toasting the succulent nut where hundreds of less imaginative manufacturers have been content to roast them.”^62^ The source of this inventive step was the subject of myth, including one tale of mechanical malfunction where an oven failed to reach its set temperature. Regardless of actual origins, the standard method was quite simple. “The nuts were first roasted sufficiently to loosen the red skins in order that they might be blanched,” Huston explained in a letter. “Then, after the blanching process they were dipped in boiling cocoanut oil to finish the cooking process.” For the final step, the hot Spanish peanuts were “cooled as quickly as possible and then the salt was added.”^63^ Tom’s took a special interest in figuring out a way to eliminate this final step, for the oily coating, which guaranteed that salt stick to the kernel, dissatisfied his imagination.

Huston identified two reasons why people did not consume more peanuts. He proclaimed to have solved the nut’s first problem by ensuring the product stay crisp “almost indefinitely.” (A claim to which I will return shortly.) Huston appealed for help with the second reason, which he described as the need to “wash your hands to remove the oil and salt after you have finished eating.”^64^ What Huston wanted, and for which he was willing to pay, was a chemical formula or a method of applying salt to peanuts without first coating the kernels in oil. He believed that impregnating peanuts with salt would give Tom’s trademark an untouchable reputation on the marketplace.

## A War within the Supply Chain

Peanuts were far from the first agricultural commodity in the United States to adhere to a strategy of mass marketing. Citrus was first. The historian Douglas Sackman has recounted the “near omnipresence of orange images” displayed by the pioneer marketing campaign for Californian citrus.^65^ Whereas other agrarian organizations in the country recoiled from the idea of consolidated industrial management, citrus growers in southern California actively adopted it. The growers’ decision to form a cooperative came in response to the monopolistic behaviour of fruit packers. All produce had to pass through the packinghouses. Patents for the latest methods of separating frost damaged goods, sizing fruit, and conveying citrus put packers in a strong position to set prices and force growers to sell on consignment.^66^ In response, the California Fruit Growers Exchange subsumed the marketing, packaging, and shipping of grower-owned fruit under a consolidated business structure with its own brand label: Sunkist.

The West Coast forerunner set the organizational model for other cooperatives, including that for peanuts. The Virginia-Carolina Cooperative Peanut Exchange formed around 1920 as an institutional pushback against the cleaners and shellers, who “controlled about 90 per cent of the annual production of ‘Virginia type’ peanuts.”^67^ With 5,000 members, the Peanut Exchange received advice directly from the California Fruit Growers Exchange. “The difference between the exchanges is largely this,” a Virginia newspaperman wrote in 1922, “the fruit growers can pack their fruits and … send them direct to the distributor, while the peanuts must undergo a very complex milling process.”^68^ The statement was only partly true, but the extra step in manufacturing peanuts granted greater leverage to the sheller.

The Peanut Exchange handled over thirty-percent of the peanut crop from the Virginia-Carolina region in 1921. That same year, Congress cleared the way for the cooperative to expand its services. Legislative changes under the 1922 Capper-Volstead Act exempted agricultural cooperatives from being treated as trusts under the law.^69^ The new legislation meant that the Peanut Exchange could clean and market its members’ peanuts under a private label. With the green light of federal approval, the Peanut Exchange reincorporated as the Peanut Growers Association in 1922.^70^

In October that year, *The Peanut Promoter* reported that Planters made “the largest single purchase” from the Peanut Growers Association.^71^ Planters, competing with five other major buyers in the region, purchased roughly a third of the total production of Virginia peanuts.^72^ The cooperative refused further sales, and with its new powers, took steps to deliver a product directly to retail. Reports from Suffolk revealed that a “brand name and trade-mark were adopted and stamped on the bags and cartons of peanuts packed by the Association.”^73^ Growers retained ownership of their harvest and now sought to capitalize on this control by advertising directly to consumers through a private label, thereby competing with the likes of Planters.

The experiment did not last long. The Association chose the name “Pickaninny Peanuts” and opted to sell their product in one-pound packages. Neither the racist colloquialism or hefty portion size won public support. According to one person involved in the campaign, the product quality was beyond rebuke, but the label suffered from obscurity and too-little advertising.^74^ In this case, Virginia growers followed Planters’ example by operating capital infrastructure for shelling peanuts and applying their own trademark to a peanut label, but their attempt to exert control within the supply chain failed to reach the consumer, illustrating a shortcoming of the brand. Establishment of a product narrative depended upon more than point-of-sale sloganeering and trademark protection.

All parties in the peanut industry did not appreciate the anticompetitive activity of the cooperative.^75^ In September 1923, peanut buyers stood trial in a federal court for inciting growers to renege on their contracts to the Peanut Growers Association. A Virginia newspaper described the case as a “so-called war between the growers and the shellers.”^76^ Per the nature of cooperative marketing agreements, which were gaining popularity in the United States at this time, members bulked their produce to limit total trade and achieve higher sale prices. Protection, in other words, came at the expense of the opportunity to sell elsewhere. A farmer might be able to earn more by foregoing the collective agreement. When commodity prices went skyward, brokers and agents looked for ways to void the terms of contract and negotiate a better price through private treaty. Such were the accusations in the federal case.

The Peanut Growers Association brought charges against fifteen shelling firms for soliciting direct sales and “circulating false reports” about market activity.^77^ Counsel for the plaintiff produced suitcases of evidence showing that the shellers had “attempted to dominate the peanut trade” and exact revenge on the growers’ cooperative.^78^ The president of Planters was put on the stand as a witness during the trial. Obici supported accusations against the shellers, alleging that they had tried to undermine growers’ prices in the past.^79^ (He claimed to have disassociated with them for that very reason.) Growers were aggrieved by the shellers’ attempt to rebalance the terms of trade.

Ultimately, the court awarded the Peanut Growers Association payment to cover its attorney fees, but also “absolved the defendants for any intentional wrong-doing.”^80^ The outcome indicated that peanut growers could try to manipulate commodity prices by collectively withholding stocks from the market, but shellers risked violation of antitrust law by colluding to set prices.^81^ Shellers may have won a battle in this so-called war, though neither sector could fully usurp the other. Exerting command over exchange relations within the agricultural supply chain required control of produce and infrastructure—both physical and informational. Tom’s, like Planters, excelled in this regard.

The historian Linda McMurry stated that the Tom Huston Peanut Company was “a buyer of peanuts, not a supplier.”^82^ In truth, Tom’s was both. Tom’s purchased its supply of peanuts from growers in the Deep South region, offering cash rewards for the highest-yielding stocks.^83^ The company also stockpiled carloads of seed to sell, at cost, back to its suppliers, an example of increased vertical integration in the peanut supply chain.^84^ The company advertised “fresh shelled white Spanish peanuts ready for planting” each Spring.^85^ The 75-pound burlap sack, replete with company logo, contained the recommended amount of seed for planting one acre. This particular detail—that the shelling division at Tom’s was selecting and redistributing Small White Spanish peanuts to conform the variety to its own quality standards—demands a reconsideration about the historical role of food processors in the seed sector.^86^ The brand label accompanied the movement of peanuts across the food supply chain.

## In the Patented Bag

**Figure 2:**
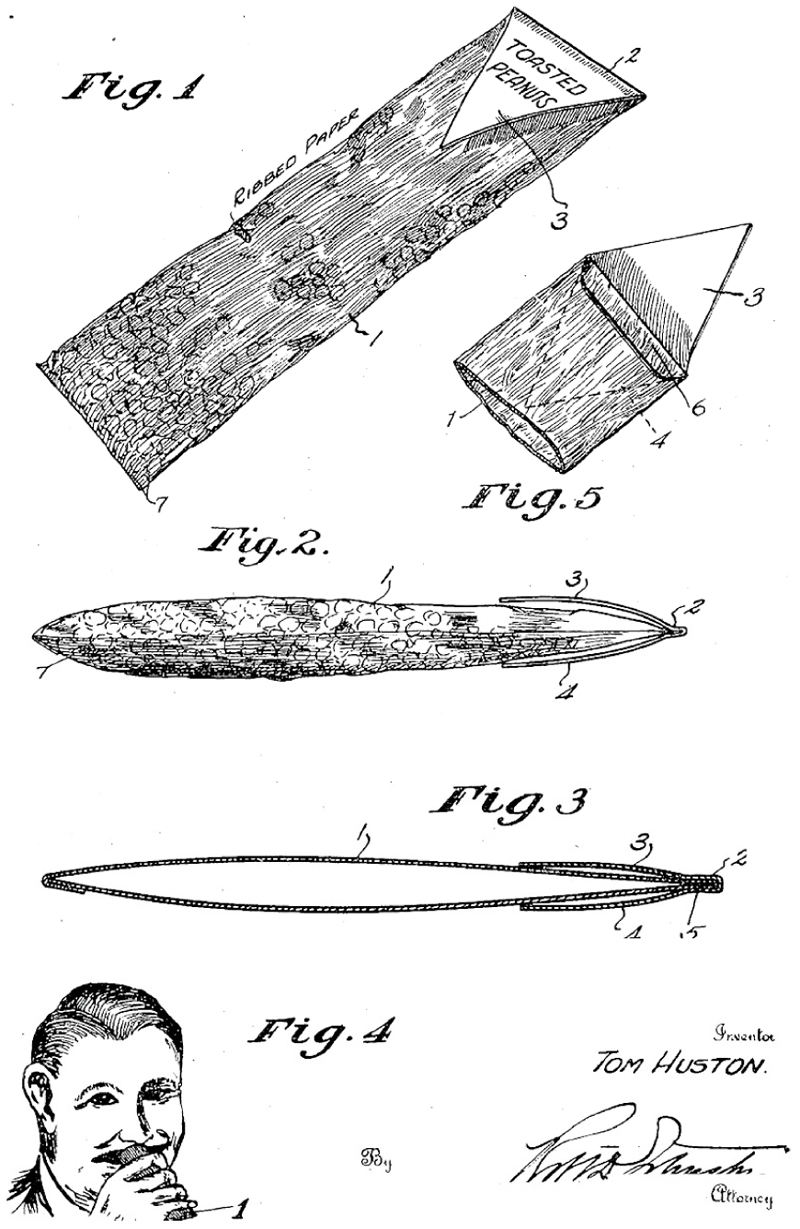
Illustration from Huston’s patent application for “Paper Bag and Seal” Source: *US Patent No. 1603207* filed on June 9, 1925 (Granted on 12 October 1926).

The founder of Tom’s wanted a chemical formula for impregnating peanuts with salt. Instead, he achieved the objective with a patented bag. In June 1925, Huston filed a patent application for a “Paper Bag and Seal” and claimed rights to a bag “longer than the width of a man’s palm” yet narrow enough to insert into the mouth.^87^ [see Fig 2] Not only did the package enable salted peanuts to be eaten without handheld contact, it kept the content crisp and tasting fresh. As will become clear, Huston’s proprietary retail bag transformed how peanuts were packaged, distributed, and consumed in modern America.

**Figure 3:**
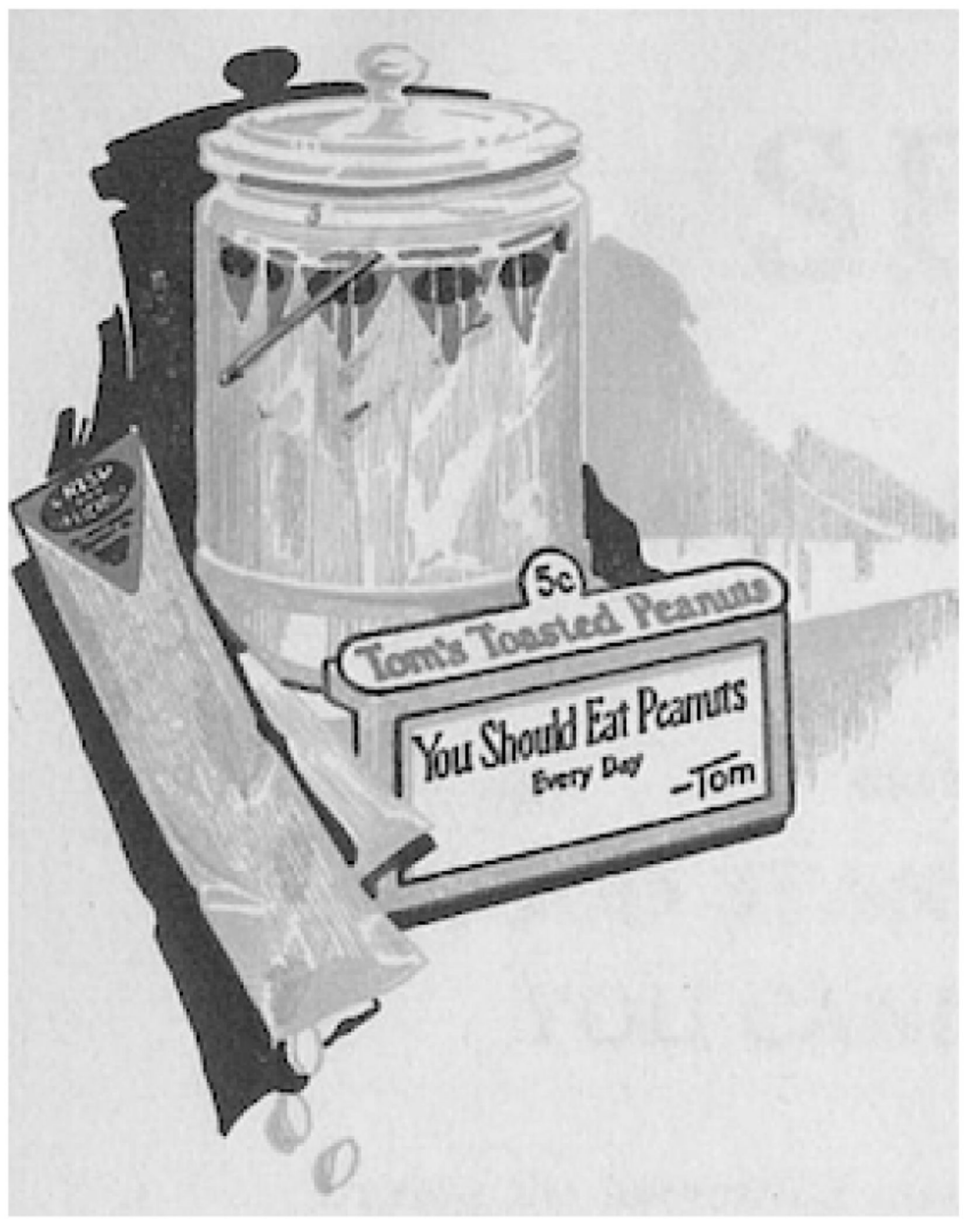
Letterhead depicting Tom’s countertop marketing material. Source: Image courtesy of The University of Georgia Libraries.

Before the end of 1925, Huston filed for another patent, this time for a peanut display stand. The patented bag and stand were designed to go together. Tall, skinny sleeves of Tom’s Toasted Peanuts stood upright in a glass jar, mounted on an eye-catching bright red plastic base. The base was cast with a countersunk flange that allowed it hold “an attractive five color lithographed poster,” which Tom’s regional salesmen changed each month.^88^ Each poster broadcast a company slogan. Huston was credited with devising the catchiest one-liners, including “They make hunger a joy.”^89^ [See Fig. 3] Placed on retail counters, right beside the cash register, the display grabbed customers’ attention at the most impulsive moment.

Tom’s advertising manager was a Georgia Tech graduate by the name of Tucker Wayne. “During Mr Wayne’s connection with the Tom Huston Peanut Company,” *The Atlanta Constitution* reported, “the concern grew to be the world’s largest packers of Spanish peanuts.”^90^ Wayne made a point to frequently remind the public about Tom’s proprietary packaging. In one newspaper advertisement from June 1929, Tom’s reprinted a Western Union telegram sent from the top of Pike’s Peak in Colorado. The sender, Bill Williams, exclaimed “I have won!” Admitting that others may ridicule his accomplishment—that of pushing a peanut up a mountain with one’s nose—Bill credited “the extra energy [he] received from eating plenty of Tom’s Toasted Peanuts every day” for the historic achievement. Tom Huston printed a personal note beneath the telegram. At first, the undertaking had struck him as “a ridiculous thing to attempt,” yet he soon decided better, knowing that Spanish peanuts would provide the wherewithal for any altitude. Huston likened his own ventures to that of the odd bod mountaineer and described himself as “just another ‘Peanut Pusher.’” The only difference, of course, was that Huston pushed peanuts all over the United States in “tall, slender bags with a red seal at the top.”^91^

Tom’s ascent suggests a plausible addendum to Gorman’s position on the historical importance of legal changes vis-à-vis personal careers in the development of visual branding. Focusing on a single domain of intellectual property overlooks the way trademark worked in conjunction with other domains of intellectual property. The fame of Tom’s trademark red triangle depended on the legal protection of the patented bag. Similarly, Planter’s trade secret added to the goodwill afforded to Mr. Peanut. An exclusive emphasis on trademarks in brand development disregards how intellectual properties interact, both with each other and material goods. In this case, trademarks worked to extend the protectability of a brand that was formed in concert with a patent or trade secret.

## Tom’s Goes to Court

Tom’s experience sizable success with the Small White Spanish peanut. In the aftermath of a fire that set his workspace ablaze in 1928, Huston built a new brick factory in Columbus to accommodate his hundreds of employees. The Tom Huston Peanut Company incorporated later that year and quickly sold-out 4,000 shares of preferred stock at $100 per share.^92^ By 1929, the company was selling $2,500,000 of peanuts a year.^93^ Huston made each district salesman into an owner. The organizational strategy paid off. In 1930, Tom’s became the first company “devoted entirely to peanut” to be listed on the New York stock market.^94^

As trade journals printed the tale of Tom’s success, other entrepreneurs took notice. A few also took to selling peanuts in lookalike bags. Huston immediately filed suit to protect his intellectual property. In February 1930, the Fulton County superior court of Georgia granted Tom’s an interlocutory injunction that stopped the Capital City Tobacco Company from vending salted peanuts in a bag resembling Tom’s own.^95^ Then, the following month, a district court in Florida upheld the validity of Tom’s patent in a decision against O. K. Jelks and Sons.^96^ Several peanut companies decided to aid Jelks in filing an appeal, Planters foremost among them.

A Circuit Court of Appeals reviewed the Jelks case in August 1930. The same court had already “had the occasion to consider the patent” in a prior case and “held that it was prima facie valid.”^97^ In that earlier case, *Huston v Barrett*, the defendant had been denied a territorial license to sell Tom’s Toasted Peanuts. Undeterred, the Barrett Potato Chip Company began to market peanuts in a bag exactly like Tom’s. The Circuit Court of Appeals reversed a trial judge’s dismissal, while admitting no certainty as whether the bag embodied the claims made in the patent.^98^

The case was escalated, and the Supreme Court of Georgia found the Barrett Potato Chip Company guilty of unfair competition. The judges concluded that the entire ensemble of Tom’s packaging had “come to denote the origin of the product.”^99^ The higher court found Barret guilty of passing off.^100^ It reinstated the injunction against Barrett’s, which allowed Barrett to sell peanuts in elongated bags, just not constructed of the same material as Tom’s. Huston was “entitled to the *combination* exclusively” and the packed product could not be imitated in its entirety without public deception.^101^

As with *Huston v Barrett*, the question put before the District Court in the Jelks case was whether to uphold the injunction and stop the company from selling salted peanuts in a bag the same size and material as Tom’s. Emboldened by its recent victory, Tom’s lawyer “filed a cross-appeal on the question of unfair competition,” which the Court of Appeals dismissed.^102^ *O K Jelks & Son v Tom Huston Peanut Company* would be judged as a patent infringement. Jelks’ counsel attempted to overturn Tom’s patent by showing that the invention had been anticipated by Planters.

Witnesses for the defense testified that, starting in 1910, Planters had sold over ten thousand boxes of salted almonds each month. The boxes, made in-house by Planters, were divided by internal compartments, “each containing two transparent bags.” Jelks’ counsel argued that these bags—being over twice as long as wide—discredited Huston’s patent. An inability to produce physical samples of the bag irritated the judges, and four copies of a trade catalogue showcasing Planters’ bagged almonds did not satisfy the legal standard for prior art. In a last effort, the defendants tried to submit that Obici’s patented double-bag from 1908 invalidated Tom’s patent. The bid proved baseless.

The Court of Appeals upheld Tom’s patent in the Jelks case, yet one judge disagreed with the verdict. Again, the issue returned to the scope of invention. The dissenting judge stated that manufacturers generally agreed that the “cheapest way to make a small paper bag” was to make it flat. For this reason, Tom’s could not claim novelty for that aspect. Nor could the use of transparent paper pass as a patentable idea. “There is therefore no possible novelty left,” the judge concluded, “but the idea of making a bag small enough to be grasped by the hand and to go into the mouth,” a form of consumption as old as the bottle itself. Consequently, the patent seemed “to rest on mere dimension,” while providing none. Such an unspecific application was outside the most liberal scope of patentability.^103^ Jelks wanted a rehearing in the Circuit Court of Appeals. The petition was denied.^104^

One detail in the case revealed how Tom’s had changed the industry with a simple inventive step. Salted peanuts in the early 1900s were either packed loose or in square paper bags. If bagged, these items were placed, flat or sideways, inside tin canisters and tended to burst in transit, resulting in losses. Tom’s, too, shipped peanuts in canisters, but because of the patented bag, the company was able to pack each item vertically.^105^ The sealed joinery of the elongated bag held-up under stress and did not break, a feat governed by the dimensions of the design. The utility of the bag enabled nationwide distribution without jeopardizing product quality. Sales skyrocketed after Huston obtained the utility patent. While the bag alone could not be held fully responsible for Tom’s sudden growth, the company wed its trademark to the unique packaging. This combination transformed the retail landscape for salted peanuts in the United States.

The historical importance of intellectual property to the distribution of agricultural products is generally subsumed within larger narratives of technological change. For instance, Gabriella Petrick showed that iceberg lettuce from California became available all year round in distant markets following World War I.^106^ The Grower-Shipper Vegetable Association of Central California organized in 1930 to dictate shipping rates for the largest packinghouses. The Association—composed of “lettuce barons” with capital equity in shipping firms, ice houses, and packing sheds—sought “to reduce the power of the large vegetable marketing firms” in eastern cities.^107^ In the late 1940s, steam-driven vacuum cooling was proven at commercial scale, boding to replace ice as a cheaper way to regulate the temperature of packed lettuce on east-bound trains. Petrick noted that, much like the “previous foray into ice manufacturing, once vacuum cooling was clearly the dominant technology”, a lettuce baron acquired the patent and restored power in the industry to the grower-shippers.^108^ Whomsoever owned exclusive rights in the method of distribution could exert demands on other actors within the supply chain. As with lettuce, so too peanuts.

Thanks to Planters’ sponsorship, Jelks continued to try to overturn the decision on the patentability of Tom’s bag. In December 1931, Jelks appealed to the Supreme Court of the United States for a writ of certiorari.^109^ Tom’s counsel responded by lodging a brief to address two unsubstantiated allegations about the patented bag. The first point was about the “indefiniteness” of its specifications. Since the description was sufficient to permit manufacture, Tom’s attorney saw no further interpretation as being necessary. Replying to the second concern, a “lack of invention”, he argued that Tom’s bag combined elements in a way that improved upon prior art. That it did so was undeniable. The counsel gushed, “*no bag ever accomplished these results before*.”^110^ Other peanut packers had illegally replicated the patented bag for this very reason.

By 1931, Tom’s had made a settlement or placed an injunction on thirty-two peanut dealers.^111^ The bag’s basic specifications did not disqualify it as an invention, but rather upheld the limits of patentability, even if the “outermost limits.”^112^ The Supreme Court refused to review the judgment.^113^ Tom’s patented bag could not be copied. It could only be improved upon.

## Prepackaged Peanuts

Failure to overturn the patent on Tom’s bag did not stop Planters from pursuing novel packaging ideas. Obici and Peruzzi were considering dispensing with the bag altogether. After all, people had been serving roasted peanuts in the shell for centuries, and Planters had the advantage of the large-podded Virginia peanuts. There was one obvious benefit to packing peanuts in a bag. The bag had surface-area for branding. Planters had become the largest peanut manufacturer in the world through the constant display of its iconographic brand. Selling peanuts without packaging would deprive the company of this distinct marketing advantage. It was preposterous to think that Tom’s glassine bag could be countered by a return to plain old, unshelled peanuts.

In 1929, Amedeo Obici filed for another patent. He secured rights for a method of applying a trademark or logo directly to a peanut shell.^114^ By conveying unshelled peanuts to a set of inking rollers, the device printed a mark directly across the irregular surface of the oblong pods “without breaking the hull.” Each individual Virginia peanut could be transformed into a branded package, voiding the need for convoluted baggage. When it came to selling peanuts in a branded pod, the lesser size of the Small White Spanish could not compete with the Virginia peanuts. The patented technology presented a way to erode the limited monopoly granted to Tom’s by making the bag outdated.

Outside the courthouse, Tom’s was busy addressing some missteps of its own. Huston had apparently not solved the problem of keeping peanuts crisp “almost indefinitely” with his patented bag. In February 1930, the Vice President of Tom’s wrote to the industrial chemist Charles Herty for advice. Herty was involved in the Chemurgic movement, applying chemistry to the manufacture of new products from agricultural crops.^115^ Tom’s asked Herty to investigate what might be causing Tom’s Toasted Peanuts to go rancid. The company hired Herty as a consultant, and he immediately began testing various materials involved in the packing and shipping of the product.^116^ Tom’s shipped five million cartons each year, so each decision on material sourcing was pricey.^117^

Herty’s initial experiments implicated the bag in imparting a sour, musty smell to Tom’s peanuts. Further findings confirmed that physical contact between the salted Spanish peanuts and wax paper resulted in oil spots. The airtight tin cartons used to ship Tom’s Toasted Peanuts were releasing a noticeable odour upon opening because of spoilage. Tom’s either needed a new type of packaging or less oily peanuts. Because the company owed its success, and that of its brand, to the Small White Spanish peanuts, Herty suggested that Tom’s consider using cellophane by DuPont, as “used on the Camel cigarette packages.”^118^ He noted, however, that in doing so, substitution of wax-paper with cellophane could cause Tom’s to reconsider of how it applied labels to packaged peanuts. Herty wanted to submit all inks and gums for further testing, including those used to adhere the trademark red triangle. In this sense, labels were not only useful in denoting content, but, as Xan Frohlich argued, the added information reformed the nature and circulation of goods. Intellectual property partook in the infrastructure for delivering processed foods.

Tom’s ended its relations with Herty in January 1932 due to severe economic constraints.^119^ With Herty gone, the founder continued to secure intellectual property protection to improve his patented bag. The USPTO granted Huston patent rights to a bag-filling apparatus in May 1932. The invention covered machines that were designed to apportion “material having a tendency to adhere to and clog,” like salted peanuts.^120^ The then-current practice for preventing the accrual of salt on packing machinery involved the manual action of brushes and scrapers. Tom’s new bagging apparatus dispensed with the need for archaic tools by incorporating a heating element. Delivery of the right amount of heat kept salt-coated peanuts free from glomming to the machine. The machine was also equipped with a nozzle for “directing an air blast downwardly into the bags.”^121^ With a combination of heat and air, the apparatus discharged the right portion of warm peanuts into an open bag. More improvements to the bag were soon to follow.

In November 1932, a patentee from Columbus, Georgia assigned Tom’s the exclusive rights to a bag seal. What this patent claimed was an origami-like method of folding bags made of cellophane. Ordinary adhesives did not perform particularly well with cellophane. To redress the foibles of prior art, including Huston’s own patented bag, the inventor disclosed a method for how “to seal the bag substantially air tight” without pasting flimsy material together.^122^ The seal did away with weaker binding, while not sacrificing the bag’s ability to “bear advertising.”^123^ The contents and dimensions of Tom’s product, combined with a limited monopoly on methods for filling and sealing the bag, set it apart. Neither Planters vacuum-sealed tins, nor their *au natural* branded pods, assembled a company trademark quite like Tom’s bag.

## Concluding Remarks

This article has illustrated how both peanut manufacturing companies, Planters and Tom’s, used trademarks in concert with other intellectual property to develop their brands. What is more, the biology of peanut types influenced how intellectual property domains combined in the commercial packaging and mass marketing of labelled products. The large Virginia-type peanut gave Planters a distinct visual advantage, one reinforced by its popular logo and secret processing method. Similarly, Tom’s delivered Small White Spanish peanuts to retail countertops nationwide through the combined protection of its bag, stand, and trademark. Branding was a package deal.

Planters remained a dominant firm in the peanut trade, regardless of Tom’s exclusive rights to the patented bag. Again, advertising was key. Planters’ gross sales expanded from $7,000,000 in 1924 to upwards of $10,000,000 in 1931, with the “world-famous trademark” found everywhere in the world, including through its chain of retail outlets, operated by the National Peanut Corporation.^124^ The company increased its spending on publicity despite the toll of the Depression.^125^

Tom Huston chose a different trajectory. He decided to diversify beyond the underground legume synonymous with his name. The founder crossed into the frozen fruit business and went broke.^126^ A bank took possession of Tom’s, the financial executive became the next president, and Huston relocated to Miami in “a self-imposed exile,” only to start other lucrative businesses.^127^

These examples from the early American peanut industry serve to complement Duguid’s proposal that branding exercises power *within* supply chains. As demonstrated, the Peanut Growers Association attempted to bypass food manufacturers with its release of a private retail label, although buyers were ultimately wary of the unknown brand. On the whole, supply relations were supportive as well as hostile. Planters and Tom’s made an effort to align their interests with growers through branding. Both companies advertised a commitment to regional suppliers through the sourcing and marketing of specific peanut types. This allegiance would change over time. Corporate growth and an expanded product line eventually uprooted the place-based devotion.^128^

This historical study of peanuts suggests an interesting aspect of intellectual property in agricultural supply chains, one that should be explored further with other brand labels. Increasingly, trademarks indicated the commercial source of food while dispensing with the geographical particulars of crop type on which the trademarks were popularized. An opposite trend may be in effect nowadays yet the larger point stays intact. Food processing and packaging changed the information conveyed, from the seed sown in tilled fields to the same sold salted beside the till.

## Notes

1 Cronon, *Nature’s Metropolis*; Soluri, *Banana Cultures*; Kurlansky, *Birdseye;* Freidberg, *Fresh;* Specht, *Red Meat Republic*, Petrick, “Industrial Food.”

2 Friedman, *Birth of a Salesman;* Maffei and Schifferstein, “Perspectives on Food Packaging Design”; Zachmann and Østby, “Food, Technology, and Trust.”

3 Weber, “The Cult of Convenience”; Veit, “Eating Cotton.”

4 Graydon, “Marketing Fictions.”

5 Higgins, ‘“Forgotten Heroes and Forgotten Issues.”

6 This article avoids any discussion of copyright, not because it is an unimportant domain of intellectual property, even for peanuts, but what little material I have concerning the copyright by Planters and Tom’s justifies its omission.

7 Chandler, *Visible Hand;* Strasser, *Satisfaction Guaranteed;* Godley, “Selling the Sewing Machine Around the World”; Carnevali and Newton, “Pianos for the People”. In relation to foodstuffs, see Lonier, “Alchemy in Eden”; Hawkins, “Advertising and the Hawaiian Pineapple Canning Industry”; Robinson, “Marketing Gum, Making Meanings”;

8 Duguid, “Developing the Brand.”

9 Gisolfi, *The Takeover*.

10 Schwarzkopf, “Turning Trademarks into Brands”.

11 Gorman, “The Role of Trademark Law,” 371.

12 Sherman and Chapman, “Rethinking Intellectual Property.”

13 Sexually-reproduced plants, including peanuts, did not become eligible for intellectual property protection in the United States until passage of the 1970 Plant Variety Protection Act.

14 Fu, “The Tyranny of the Bottle”; Hawkins, “The Performativity of Food Packaging”*;* Hisano, “Selling Food in Clear Packages.” Ventimiglia,‘“Deceptions Have Been Practiced”’.

15 Frohlich, “The Informational Turn”.

16 The Virginia Runner variety dominated the commercial class of Virginia peanut, but there was also a Virginia Bunch variety with semi-prostrate growth habit. Higgins, *History of the Georgia Experiment Station*.

17 Roper, *The Peanut*, 8.

18 “Virginia has World’s Greatest Peanut Market, Suffolk Boasts,” *The World News*, Roanoke, 1 December 1, 1921, 13.

19 *US Trademark Registration No. 38883* (Granted on 8 September 1902).

20 “One Way to Spell it,” *The Suffolk News-Herald*, March 17, 1937, Editorial and Features’ Page.

21 Ibid.

22 “Virginia’s Peanut Mogul,” *The Suffolk News-Herald*, Suffolk, March 12, 1937.

23 “How Advertising Put Mr. Peanut on the Map,” *The Peanut Promoter* 7, no. 1 (1923): 13.

24 “City Shares Peanut Claim,” *The Wilkes-Barre Record*, October 19, 1940, 2; “Planters’ Peanuts Very Popular,” *The Scranton Republican*, March 5, 1919, 6.

25 *US Patent No. 892074*, filed on June 10, 1907 (Granted on June 30, 1908).

26 “A Great Community Loss,” *The Suffolk News-Herald*, May 22, 1947, 6; “Planters to Open Branch in Portsmouth, Va.” *The Peanut Promoter* 7, no. 2 (1924): 13.

27 “Planters Has Grown in 35 Years from Small business to Tremendous Organization in America and Canada,” *The Suffolk News-Herald*, January 27, 1941, 2.

28 “Peruzzi Talk to Kiwanians,” *The Wilkes-Barre Record*, February 3, 1928, 3.

29 Johnston, “Building an Industry on Peanuts,” *The Smithfield Times*, April 9, 1936.

30 Koehn, “Henry Heinz and Brand Creation,” 365, 390; Petrick, “Feeding the Masses.”

31 See Elmore, “Citizen Coke,” 724.

32 Veit, “Eating Cotton”; Domosh, “Pickles and Purity.”

33 “Advertising Put Mr. Peanut on the Map,” *The Peanut Promoter* 7, no. 1 (1923): 13; “Back of all the Development of America is the Power of Advertising’,” *The Peanut Promoter* 7, no. 2 (1924): 24.

34 Newspaper clipping, “Patent on Device for Peanut Bag Held to Be Valid,” September 17, 1931, Emory University Archives, MSS 8, Box 106, Folder 16.

35 *The Complete World of Mr. Peanut*, 1967.

36 Quote from an August 1950 article cited in *Planters Nut & Chocolate Company v Crown Nut Company, Inc*. 305 F.2d 916 (CCPA 1962).

37 Franz, “Mr. Peanut and Antonio Gentile,” *American History*, May 6, 2014; Kirby, “The Life and Times of Mr. Peanut,” *Contingent Magazine*, February 13, 2020.

38 “Virginia has World’s Greatest Peanut Market.”

39 “Sights and Insights of Everyday Life,” *Suffolk News-Herald*, April 4, 1932; “Planters to Open Branch in Portsmouth, Va,” *The Peanut Promoter* 7, no. 2 (1924): 13.

40 “Advertising Put Mr. Peanut on the Map,” *The Peanut Promoter* 7, no. 1 (1923): 13.

41 Carney & Rosomoff, *In the Shadow of Slavery*, 141-143.

42 B.B. Higgins noted that this observation repeatedly gets attributed to a single comment by Hans Sloane. Higgins, “Origin and Early History”, 24; see also, “Peanut Brand by Planters Well Known” *Wilkes Barre Times Leader*, October 24, 1931, 15.

43 Roper, *The Peanut*, 7.

44 “Rise of the Peanut,” *Evening Star*, Washington, May 29, 1921, 53.

45 “The True Measure of Worth,” *The Suffolk News-Herald*, July 25, 1931, 4.

46 Advertisement, “Introducing ‘Mr. Peanut,’” *The Barre Daily Times*, October 2, 1917.

47 Ibid.

48 “How Advertising Put Mr. Peanut on the Map,” *The Peanut Promoter* 7, no. 1 (1923): 13.

49 Advertisement, “From Sunny Southern Fields,” *The Orlando Sentinel*, December 1, 1917.

50 Advertisement, “Give Me A Place at Dinner,” *The Barre Daily Times*, October 2, 1917.

51 Pollay, “The Subsiding Sizzle,” 24, 30-31.

52 *US Trademark No 121818* (Granted on May 28, 1918).

53 The indication of geographical origin served the branding strategies of other agricultural crops, even in the United States. See Olsson, “Peeling Back the Layers”.

54 Letter from Herty to Hoover, July 31, 1931, Emory University Archives, MSS 8, Box 105, Folder 15; Giesen, *Boll Weevil Blues*.

55 “Tom’s Peanuts,” *Time* 16, no. 8 (August 25, 1930), 1; “Genius in Exile,” *Sunday Ledger-Enquirer Magazine*, Columbus, August 29, 1965; Sanders, “Few Peanuts Grow into Big Business,” *The Oregon Stateman*, Salem, December 22, 1929, 19.

56 *US Patent No. 1246100*, filed on July 3, 1915 (Granted on November 13, 1917).

57 Letter from Medley to Stuckey, April 22, 1931, UGA, UA 07-022, Box 9, Folder 16; Advertisement, *The Montgomery Advertiser*, January 14, 1917, 35.

58 “Tom Huston Manufacturing Co. Incorporates,” *Peanut Promoter* 2 (1918): 37; “A Peanut Machine,” *The Peanut Promoter* 1 (1918): 29.

59 Letter from Huston to Carver, October 18, 1924, TU, Collection 006, GWC Papers.

60 *US Patent No. 1412614*, filed on May 2, 1919 (Granted on April 11, 1922).

61 “Tom’s Peanuts,” *Time* 16, no. 8 (25 August 1930): 1; Newspaper clipping, “Tom Huston, 82, Founder of Tom’s Toasted Peanuts,” CSU, MC 37, Box 1.

62 “Huston Peanut Plant Sets Progress Record,” *The Peanut Promoter* 10 (1927): 9.

63 Letter from Huston to Carver, October 18, 1924, TU, Collection 006, GWC Papers.

64 Ibid.

65 Sackman, ‘“By Their Fruits,” 82, 89.

66 Moses, “A Citrus Monopoly.”

67 “Peanut War Suit Ends in Agreement by Both Sides,” *The Peanut Promoter* 7, no. 11 (1923): 28.

68 “Peanut Growers’ Exchange Enters Cleaning Operation,” *The Times Dispatch*, Richmond, VA, March 15, 1922, 5.

69 Ibid.

70 Clay, *Marketing Peanuts*.

71 “Big Deal in Peanuts,” *The Peanut Promoter* 5, no. 12 (1922): 18.

72 “The Yellow Peril,” *The Peanut Promoter* 7, no. 3 (1924): 11.

73 “Is the Advice Sound?” *The Suffolk News-Herald*, 15 November 15, 1928, 28.

74 “Packaged Peanuts and the Retail Trade,” *The Peanut Promoter* 7, no. 1 (1923): 9.

75 “Making Fatal Mistake,” *The Suffolk News-Herald*, Suffolk, 1 March 1930, 4.

76 “Peanut Buyers Placed on Stand at Norfolk Trial,” *The Bee*, Danville, VA, September 20, 1923, 5.

77 “Alleged Peanut Conspiracy,” *The Peanut Promoter* 5, no. 12 (1922): 18.

78 “Peanut War Suit Ends,” 29.

79 “Peanut Buyers Placed on Stand,” 5.

80 Ibid 1.

81 “Daugherty Hits Association Trade Secrets.” *The Peanut Promoter* 7, no. 3 (1924): 26.

82 McMurray, *George Washington Carver*, 221.

83 “Many Prizes Won,” *National Nut News* (March 1929): 69.

84 Letter from Tom Huston to H P Stuckey, March 23, 1927, UGA, UA 07-022 Box 5, File 8; Barry and Porter, *Peanuts*.

85 Advertisement, “Plant Peanuts,” *The Atlanta Constitution*, Atlanta, May 24, 1928, 21.

86 See Sherman and Chapman, “Rethinking Intellectual Property.”

87 *US Patent No. 1603207*, filed on June 9, 1925 (Granted on October 12, 1926).

88 *US Patent No. 1603954*, filed on December 4, 1925 (Granted on October 19, 1926); Minnie Huston, “Tom Fed Peanuts to All”; “Tom Huston – Fact or Fancy,” CSU, MC 37, Box 2, Folder 19.

89 “Tom Huston – Fact or Fancy,” CSU, MC 37, Box 2, Folder 19.

90 “Tucker Wayne Joins J A Green & Co,” *The Atlanta Constitution*, March 13, 1932, 15.

91 Advertisement, *The Tennessean*, Nashville, June 12, 1929, 3.

92 “Tom Huston Company Declares Dividend”, *The Atlanta Constitution*, Atlanta, December 20, 1928, 21; “Tom Huston Issue is Oversubscribed”, *The Atlanta Constitution*, Atlanta, October 28, 1928, 40; “Peanuts Make Farm Boy Fortune”, *Lubbock Avalanche Journal*, Lubbock, TX, October 6, 1929, 8.

93 Sanders, “Few Peanuts Grow into Big Business”.

94 “Tom’s Peanuts”.

95 “Injunction Granted to Peanut Company,” *The Atlanta Constitution*, February 21, 1930, 3.

96 “Validity of Peanut Patent Sustained,” *The Atlanta Constitution*, March 13, 1930, 9.

97 *O K Jelks & Son v Tom Huston Peanut Company*, 52 F 2d 4 (Ga 5^th^ Cir, 1931).

98 *Huston v Barrett*, 23 F 907 (Ga 5^th^ Cir, 1928).

99 *Huston v Barrett*, 166 Ga 261 (Ga Sup Ct, 1928); *Barrett v Huston*, 166 Ga 261 (Ga Sup Ct, 1928).

100 In common law countries, such as the United States, unfair competition involves deception. It includes practices which create confusion with the business of a competitor, those that falsely discredit a competitor’s business, and those that mislead the public as to the characteristics of the goods being offered. In civil law countries, unfair competition has a broader scope that does not involve deception.

101 *Huston v Barrett*, 166 Ga 261 (Ga Sup Ct, 1928); *Barrett v Huston*, 166 Ga 261 (Ga Sup Ct, 1928). (italics in original).

102 *O K Jelks & Son v Tom Huston Peanut Company*.

103 Ibid (Sibley J).

104 “Decisions Denying Certiorari,” 284 US 617 (1931) 686.

105 *O K Jelks & Son v Tom Huston Peanut Company*, 52 F 2d 4 (Ga 5^th^ Cir, 1931).

106 Gabriella M Petrick, ‘“Like Ribbons of Green and Gold”, 269.

107 Ibid 276.

108 Ibid 283.

109 *O K Jelks & Son v Tom Huston Peanut Company*, No 577 (US Supreme Court, 1931).

110 Ibid, “Brief in Opposition to Petition”, 4 (italics in original).

111 *O K Jelks & Son v Tom Huston Peanut Company*, 52 F 2d 4 (Ga 5^th^ Cir, 1931) 3.

112 *Westcott Hosiery Mills v Rich’s* 56 F 2d 234 (1932).

113 *O K Jelks & Son v Tom Huston Peanut Company*, No 577 (US Supreme Court, 1931).

114 *US Patent No. 1735471*, filed on October 16, 1928 (Granted on November 12, 1929).

115 Finlay, “The Failure of Chemurgy,” 85.

116 These tests seem to have been done in cooperation with the Robert Gair Company, whose founder patented the cardboard box. Letter from Anon to Herty, April 15, 1931, CSU, MSS 8, Box 106, Folder 15.

117 Letter from Woleben to Herty, November 12, 1930, CSU, MSS 8, Box 107, Folder 4.

118 Letter from Herty to Woleben, November 15, 1930, CSU, MSS 8, Box 107, Folder 4.

119 Letter from Richards to Herty, December 27, 1933, CSU, MSS 8, Box 107, Folder 1.

120 *US Patent No. 1859562*, filed on December 14, 1929 (Granted on May 24, 1932).

121 Ibid.

122 *US Patent No. 1889001*, filed on 16 April 1932 (Granted on November 29, 1932) for a bag seal, which the patentee Belmont D Osteen assigned to the Tom Huston Peanut Company.

123 Ibid, 1.

124 “Peanut Brand by Planters Well Known”; Smith, *Peanuts*, 51-54.

125 Smith, *Peanuts*, 55.

126 “Tom Huston - Fact or Fancy,” 4; Thwaite, “Georgia Frozen Food Pioneer Failed in ‘30s,” *The Atlanta Constitution*, October 6, 1966, 63.

127 “W.A. Richards to Head Tom Huston Company,” *Atlanta Constitution*, January 28, 1932, 7; Letter from Richards to Huston, June 4,1954, MC 37, Box 2; “Genius in Exile”, *Sunday Ledger-Enquirer Magazine*, Columbus, GA, August 29, 1965, CSU, MC 37, Box 2, Folder 33.

128 Peanut oil, candy bars, and peanut butter showcased less specificity in type. For certain products, Planters did continue to market Virginia peanuts, even as the commercial class of Virginia peanuts were increasingly sourced from overseas suppliers.

## References

[R1] Carney Judith, Rosomoff Richard N (2011). In the Shadow of Slavery: Africa’s Botanical Legacy in the Atlantic World.

[R2] Clay Henry Johnson (1941). Marketing Peanuts and Peanut Products.

[R3] Cronon William (1991). Nature’s Metropolis: Chicago and the Great West.

[R4] Freidberg Susanne (2009). Fresh: A Perishable History.

[R5] Friedman Walter A (2004). The Birth of a Salesman: The Transformation of Selling in America.

[R6] Giesen James C (2011). Boll Weevil Blues.

[R7] Gisolfi Monica R (2017). The Takeover: Chicken Farming and the Roots of American Agribusiness.

[R8] Higgins Bascombe B (1975). History of the Georgia Experiment Station, 1889-1975.

[R9] Kurlansky Mark (2012). Birdseye: The Adventures of a Curious Man.

[R10] McMurry Linda O (1981). George Washington Carver: Scientist & Symbol.

[R11] Roper William N, Roper WN (1905). The Peanut and Its Culture.

[R12] Smith Andrew F (2002). Peanuts: The Illustrious History of the Goober Pea.

[R13] Soluri John (2005). Banana Cultures: Agriculture, Consumption, and Environmental Change in Honduras and the United States.

[R14] Specht Joshua (2019). Red Meat Republic: A Hoof-to-Table History of how Beef Changed America.

[R15] Wrenn Lynette Boney (1995). Cinderella of the New South: A History of the Cottonseed Industry, 1855-1955.

